# Gene expression profiles in Atlantic salmon adipose-derived stromo-vascular fraction during differentiation into adipocytes

**DOI:** 10.1186/1471-2164-11-39

**Published:** 2010-01-17

**Authors:** Marijana Todorčević, Stanko Škugor, Aleksei Krasnov, Bente Ruyter

**Affiliations:** 1 Nofima, Norwegian Institute of Food, Fisheries and Aquaculture Research, P.O. Box 5010, Ås NO-1430, Norway; 2 Department of Animal and Aquacultural Sciences, Norwegian University of Life Sciences, P.O. Box 5003, Ås NO-1432, Norway

## Abstract

**Background:**

Excessive fat deposition is one of the largest problems faced by salmon aquaculture industries, leading to production losses due to high volume of adipose tissue offal. In addition, increased lipid accumulation may impose considerable stress on adipocytes leading to adipocyte activation and production and secretion of inflammatory mediators, as observed in mammals.

**Results:**

Microarray and qPCR analyses were performed to follow transcriptome changes during adipogenesis in the primary culture of adipose stromo-vascular fraction (aSVF) of Atlantic salmon. Cellular heterogeneity decreased by confluence as evidenced by the down-regulation of markers of osteo/chondrogenic, myogenic, immune and vasculature lineages. Transgelin (TAGLN), a marker of the multipotent pericyte, was prominently expressed around confluence while adipogenic PPARγ was up-regulated already in subconfluent cells. Proliferative activity and subsequent cell cycle arrest were reflected in the fluctuations of pro- and anti-mitotic regulators. Marked regulation of genes involved in lipid and glucose metabolism and pathways producing NADPH and glycerol-3-phosphate (G3P) was seen during the terminal differentiation, also characterised by diverse stress responses. Activation of the glutathione and thioredoxin antioxidant systems and changes in the iron metabolism suggested the need for protection against oxidative stress. Signs of endoplasmic reticulum (ER) stress and unfolded protein response (UPR) occured in parallel with the increased lipid droplet (LD) formation and production of secretory proteins (adipsin, visfatin). The UPR markers XBP1 and ATF6 were induced together with genes involved in ubiquitin-proteasome and lysosomal proteolysis. Concurrently, translation was suppressed as evidenced by the down-regulation of genes encoding elongation factors and components of the ribosomal machinery. Notably, expression changes of a panel of genes that belong to different immune pathways were seen throughout adipogenesis. The induction of AP1 (Jun, Fos), which is a master regulator of stress responses, culminated by the end of adipogenesis, concurrent with the maximal observed lipid deposition.

**Conclusions:**

Our data point to an intimate relationship between metabolic regulation and immune responses in white adipocytes of a cold-blooded vertebrate. Stress imposed on adipocytes by LD formation and expansion is prominently reflected in the ER compartment and the activated UPR response could have an important role at visceral obesity in fish.

## Background

Feeds used in modern Atlantic salmon aquaculture contain large amounts of lipids, which provide rapid growth, reduce environmental load from farms, but increase visceral fat deposition [1]. Very little is, however, known about the factors regulating development and functions of adipose tissue in fish, and whether increased fat deposition may lead to health problems. Previously regarded principally as energy storage, white adipose tissue (WAT) in mammals is now recognized as a highly active endocrine tissue producing numerous secretory proteins, including adipokines, a suite of small signalling proteins specifically produced in WAT [2,3]. Under normal conditions, adipocytes are involved in the regulation of a broad range of physiological processes but at obesity increased production of cytokines and adipokines lead to the chronic low-grade inflammatory state. The extent of conservation of endocrine WAT functions in the cold-blooded vertebrate, including its capacity to influence systemic inflammatory responses and development of life style related disorders has not been studied until now.

In salmonid fish, precursor cells differentiate into adipocytes continuously [4], however, the exact origin of preadipocytes is not known. In mammals, development of white adipocytes is thought to begin with progenitor cells from the perivascular compartment (pericytes) in WAT, characterised by expression of smooth muscle actin 22α, also known as transgelin (TAGLN), and other vascular markers. Isolated pericytes differentiate into mesenchymal stem cells (MSCs), which in turn, give rise to various cell types including osteoblasts, chondrocytes, smooth muscle cells, fibroblasts, macrophages and adipocytes [5-9]. Mammalian adipose stromal-vascular fraction (aSVF) harbours a population of progenitor cells that is also capable to differentiate *ex vivo *into cells and tissues of mesodermal origin, thus suggesting their perivascular origin. Atlantic salmon WAT contains a large number of fibroblast-like precursor cells that can differentiate to mature adipocytes *in vitro *[10,11], however, their stem cell features have not been characterized yet.

In mammals, adipogenesis includes three distinct stages. Following active proliferation and the phase of determination, cells reach confluence, followed by hormonal induction and terminal differentiation. Re-entry into the cell cycle of growth-arrested cells at confluence involves several rounds of proliferation, referred to as mitotic clonal expansion. Secondary growth arrest and induction of the transcription factors CCAAT-enhancer-binding protein (C/EBP) α and peroxisome proliferator-activated receptor (PPAR) γ mark the end of the mitotic clonal expansion phase and entry into terminal differentiation with transcriptional activation of genes defining the mature adipocyte phenotype.

The few performed studies indicate that adipogenesis in fish bears many resemblances to that of terrestrial vertebrates [10-12], though there certainly exist as yet undiscovered species-specific differences. Atlantic salmon precursor cells take longer than mammalian to acquire the mature adipocyte phenotype in culture, due to low incubating temperature, typical of salmonid fish habitat. Further, mammalian cells are able to produce lipid droplets (LDs) from glucose alone, while salmon preadipocytes require lipids in order to achieve the mature phenotype [10]. Hence it is to be expected that white adipocytes and their precursors in a cold-blooded vertebrate may have both evolutionarily conserved and specific features.

The aSVF primary culture enabled investigation of early events involved in the adipogenic determination as well as processes characteristic of the later terminal differentiation phase. Particular emphasis was placed on genes governing nutrient metabolism and stress and inflammatory responses.

## Results

### Cell culture characterization

**Figure 1:**
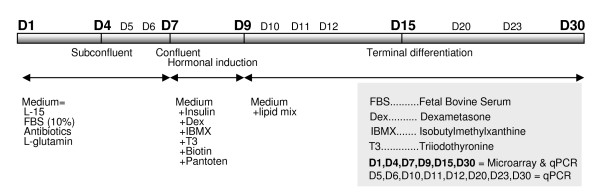
**Summary of procedures and the key stages**.

**Figure 2:**
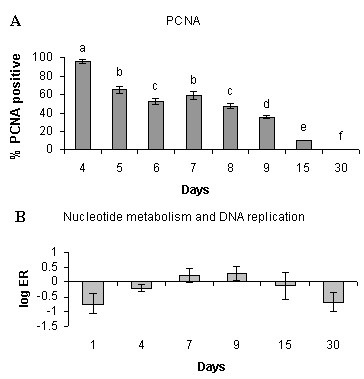
**Cell division, DNA replication and nucleotide metabolism**. **A**: cell proliferation was assessed by immunocytochemical detection of proliferating cell nuclear antigen (PCNA). Data (labeling index) are shown as mean ± SEM (n = 5). Different letters indicate significant differences (p ≤ 0.05). **B**: expression changes (mean log-ER ± SE) of eight genes (Cdc45, cell division control protein 42 homolog, anti-silencing function 1B, ribonucleoside-diphosphate reductase large subunit, cold inducible RNA binding protein-1 and 2, chromosome segregation 1-like protein, chromosome-associated kinesin KIF4A) analyzed with microarrays.

**Figure 3:**
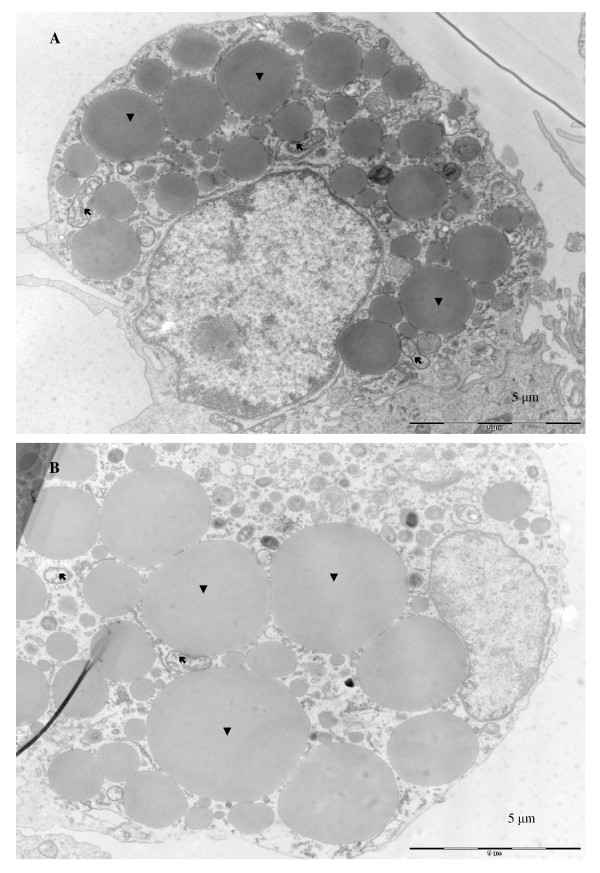
**Atlantic salmon aSVF primary cell culture**. **A, B**: Electron micrographs of representative salmon adipocytes day 15 (A) and day 30 (B). Three weeks after induction of differentiation (day 30), a large portion of cytoplasmic space was filled with LDs, the number mitochondria was reduced and the nuclei were located between LDs and cell membranes. Bars: 5 μm. Arrows points to mitochondria and arrowheads to lipid droplets

**Figure 4:**
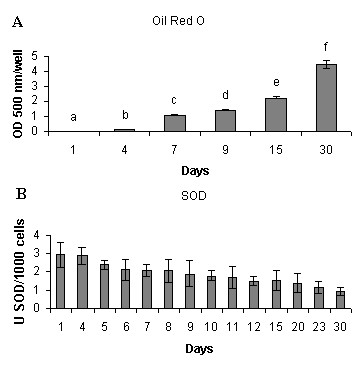
**A: lipid content in adipocytes was quantified with Oil Red O. **Absorbance of extracted dye was measured at 500 nm. Data are shown as mean ± SEM (n = 5). Different letters indicate significant differences (p ≤ 0.05). **B**: superoxide dismutase (SOD) activity in culture media. Different letters indicate significant differences (p ≤ 0.05). Data shown are mean ± SEM (n = 5).

A short summary of adipocyte development and cultivation conditions is presented in Fig. 1. In the subconfluent stage during the first seven days of culture, aSVF cells had the highest proliferative activity (Fig. 2A). Approximately at day 7, cells reached the confluent stage. Two-day adipogenic hormonal induction was applied at that time point. This was followed by the terminal differentiation stage during which cells acquired a more rounded shape. Morphological changes observed by electron microscopy images at days 15 and 30 were characteristic of the terminally differentiating phenotype of adipocytes, including a relatively low mitochondrial number, large size of LD and the nucleus located between LDs and cell membrane (Fig. 3A, B). Oil red O staining also showed a high degree of lipid accumulation in mature adipocytes at day 30 (Fig. 4A). A steady decrease in the extracellular superoxide dismutase (SOD) activity was observed from day 1 to day 30 (Fig. 4B).

### Subconfluence (days 1 and 4)

**Figure 5:**
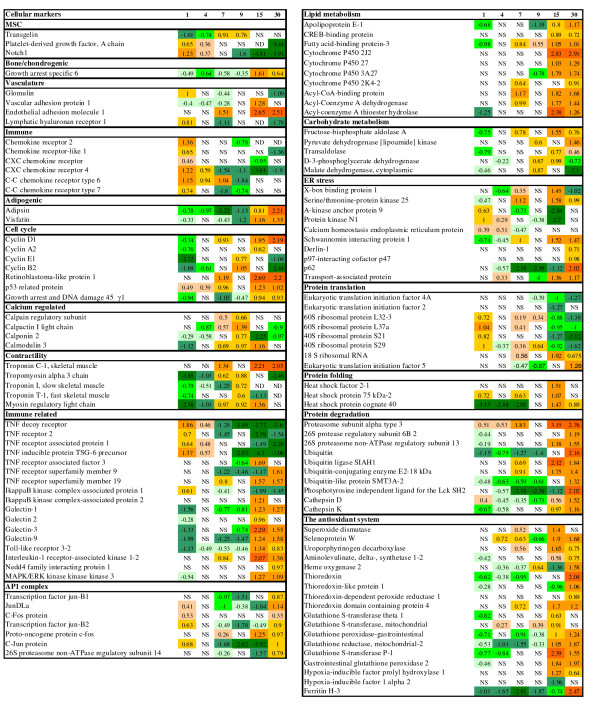
**Results of microarray analyses, selected genes with expression changes**. Samples from days 1, 4, 7, 9, 15 and 30 (5 pooled flasks per day) were compared to an equalised mixture from all time-points. Data are log-ER (expression ratios). Significantly up- and down-regulated genes (p < 0.01, t-test, 12 spot replicates per gene) are highlighted with red and green scales. NS - not significant, ND - not detected.

**Figure 6:**
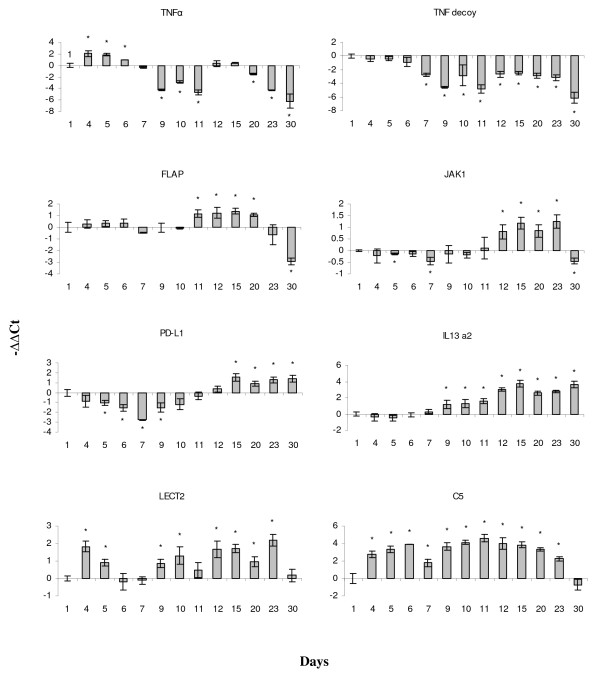
**Immune genes analyzed with real-time qPCR**. In this and other panels data are shown as -ΔΔCt ± SE (n = 5). Significant difference from day 1 (t-test, p < 0.05) is indicated with *. TNFα - tumour necrosis factor alpha, TNF decoy - tumour necrosis factor decoy receptor, FLAP - 5-lipoxygenase-activating protein, JAK1 - janus kinase 1, PD-L1 - programmed cell death ligand 1, IL-13 a2 - interleukin 13, LECT2 - leukocyte cell-derived chemotaxin a2, C5 - complement component 5.

**Figure 7:**
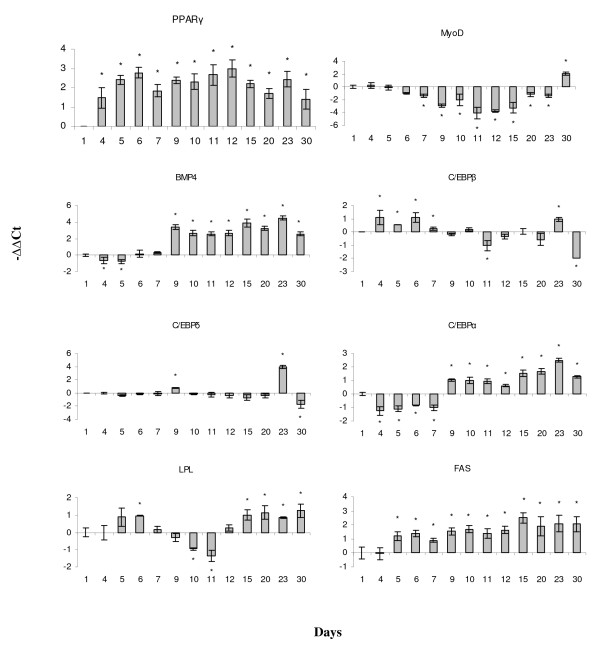
**Cell lineage markers analyzed with real-time qPCR**. PPARγ - peroxisome proliferator-activated receptor γ, MyoD - myogenic factor D, BMP4 - bone morphogenic protein 4, 1C. C/EBP - CCAAT/enhancer-binding proteins α, β and δ, LPL - lipoprotein lipase, FAS - fatty acid synthase.

Microarray analyses of aSVF in the first days of culture, revealed concurrent expression of genes characteristic of MSCs, immune cells and cells of perivascular origin (Fig. 5). Platelet-derived growth factor A (PDGFA) is a major regulator of proliferation and migration of mammalian adipose-derived MSCs [13]. Glomulin is essential for normal development of the vasculature while the lymphatic vessel endothelial hyaluronan receptor 1 (LYVE1) is a marker of lymph vessels and endothelial cells in mammals. Chemokine receptor 4 (CXCR4) and other chemokine/cytokine receptors are involved in cell mobilisation and retention in several populations of MSCs and in immune cells [14-16]. We have selected eight co-expressed genes encoding proteins involved in nucleotide metabolism, DNA replication and regulation of cell cycle and their averaged profile, due to their involvement in the similar cellular functions, was shown in Fig. 2B. The lowest expression of genes from this category was at day 1 (also see Fig. 5). The subsequent increase was in concordance with the results of proliferating cell nuclear antigen (PCNA) assay that revealed the highest proliferation rate at day 4 (Fig. 2A). In parallel, microarray analyses showed a decreased expression of non-adipogenic cell markers. These genes had high levels at day 1 (Fig. 5). Tumour necrosis factor (TNF) α, a proinflammatory cytokine and a potent negative regulator of adipocyte differentiation in terrestrial vertebrates, was up-regulated before confluence as well as a panel of TNF-related genes and receptors (Fig. 5 and 6). We observed up-regulation of PPARγ already at day 4 (Fig. 7). This is the master regulator that co-ordinately activates transcription of adipocyte-specific genes [17,18].

### Confluence (day 7) and hormonal induction (day 9)

Cells reached confluence at day 7. Up-regulation of retinoblastoma-like protein 1 (RBL1) and p53-like protein (Fig. 5) was in concordance with the temporary cell cycle arrest at G_0_/G_1_, which is commonly observed at this stage in most mammalian cell lines. After stimulation with hormones, cells re-enter the cell cycle and undergo mitotic clonal expansion before ultimate exit from the cell cycle. At day 9, expression of RBL1 and p53-like protein decreased while cyclins E1 and B2 were stimulated. Two days after addition of the differentiation media, cells changed to a more rounded shape. This coincided with the up-regulation of a suite of motor contractile proteins (Fig. 5). Interestingly, microarray showed elevated expression of TAGLN at day 7 and day 9. TAGLN is a marker of pericytes, smooth muscle-like cells surrounding vasculature, that are closely related to the MSCs and fibroblasts [19]. Positive and negative regulators of myogenic differentiation showed opposite changes: decrease in myogenic factor D (MyoD) and increase in bone morphogenic protein 4 (BMP4) (Fig.7). We also observed up-regulation of several genes for Ca^2+ ^binding proteins (Fig. 5). Calcium is involved in the control of the whole adipogenic process, from multipotent stem cells to adipocytes [20,21]. Increases in cytoplasmic calcium during this phase inhibit adipogenesis [21-24]. Days 7 and 9 were marked by the expression changes of C/EBPs. The early adipogenic marker C/EBPβ, was up-regulated until confluence, whereas transient induction of C/EBPδ and consistent increase of C/EBPα were observed after the addition of hormones at day 9 (Fig. 7). Overall, C/EBPβ and C/EBPδ work sequentially and predate the expression of C/EBPα [12]. We observed different expression changes in a number of genes encoding chemokines and cytokines. The leukocyte cell-derived chemotaxin 2 (LECT2) was re-activated at day 9 and remained up-regulated until the end of the study period (Fig. 6). LECT2 is a potent neutrophil chemoattractant, which also affects development of chondrocytes and osteoblasts [25,26]. In contrast, TNFα and related genes were down-regulated (Fig. 5 and 6).

### Terminal differentiation and the late phase of white adipocyte maturation (days 12-30)

**Figure 8:**
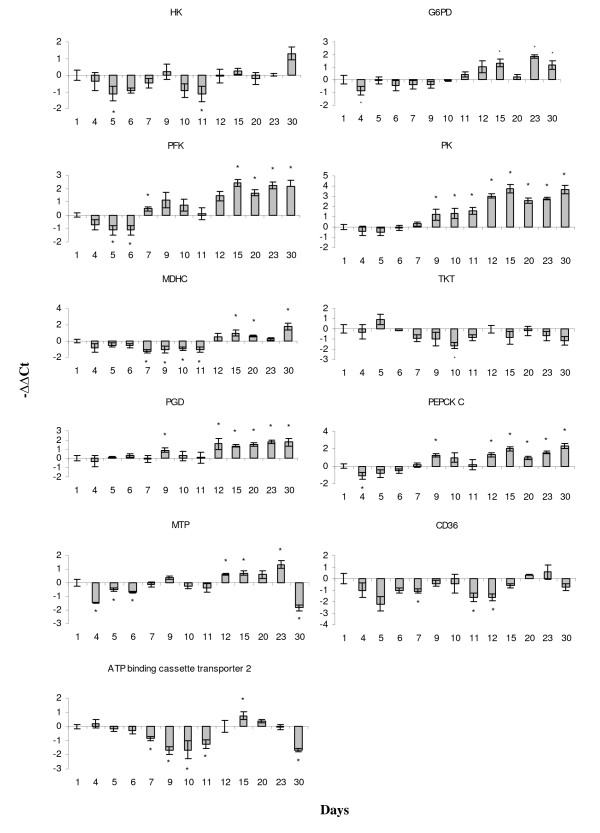
**Genes for enzymes from different pathways of carbohydrate and lipid metabolism analyzed with real-time qPCR**. HK - hexokinase, G6PD - glucose-6-phosphate dehydrogenase, PFK - phosphofructokinase, PK - pyruvate kinase, MDHC - malate dehydrogenase, PEPCKC - phosphoenolpyruvate carboxykinase C, PGD - 6- phosphogluconate dehydrogenase, TKT - transketolase, MTP - microsomal triglyceride transfer protein, CD36 - cluster of differentiation 36, ATP binding - ATP-binding cassette transporter 2.

The increase in intracellular lipid levels measured by Oil Red O (Fig. 4A) was in line with the up-regulation of genes for several proteins of lipid metabolism (Fig. 5), including lipoprotein lipase (LPL) (Fig. 7). The expression of fatty acid synthase (FAS) was highest from day 15 to day 30 (Fig. 7) in agreement with the profile of FAS observed in murine adipocytes [27]. Metabolic alterations were also supported by the regulation of key genes from major carbohydrate pathways (Fig. 8). Glucose-6-phosphate dehydrogenase (G6PD) and 6-phosphogluconate dehydrogenase (PGD) from the oxidative pentose phosphate pathway (PPP) were up-regulated from day 12 to the end of adipogenic differentiation indicating increased need for NADPH that is required for TAG biosynthesis. To synthesise nucleotides, cells need large amounts of ribose-5-phosphate (R5P). The PPP uses glucose to provide R5P and NADPH, however, the output can be modified according to the cells' current needs, towards the production of either one of the products. The increase of phosphofructokinase (PFK) and pyruvate kinase (PK) expression from day 15 could be an evidence for the importance of glycolysis in TAG-accumulating adipocytes. In addition to glycolysis and contrary to what was earlier believed, adipocytes seem to be able to use alternative means (glyceroneogenesis) to produce glycerol-3-phosphate (G3P) needed for TAG synthesis [28,29]. Two key glyceroneogenic enzymes, cytoplasmic malate dehydrogenase (MDHC) and cytosolic phosphoenolpyruvate carboxykinase (PEPCKC) were also up-regulated during terminal differentiation. These changes did not necessarily mean a switch to anaerobic metabolism. Microarray analyses revealed a decrease of hypoxia inducible factor (HIF1α) while the negative regulator of HIF1α (HIF prolyl hydroxylase) was induced (Fig. 5).

Day 15 was marked with the expression changes of genes involved in differentiation and cell cycle. Increase was observed in the anti-osteogenic [30] growth arrest specific protein 6 (GAS6) and adipogenic markers adipsin and visfatin (Fig. 5). The up-regulations of RBL1, p53-like protein and growth arrest and DNA-damage-inducible (GADD45) γ were in concordance with the marked attenuation of cell proliferation. Interestingly, many immune-related genes exhibited sustained up-regulations, which began shortly before or at the onset of terminal differentiation (Fig. 6). Programmed death ligand 1 (PD-L1) is involved in the blockage of cell cycle in T-cells [31]. The up-regulation of decoy receptor interleukin (IL) 13 receptor alpha 2 (IL13Rα2) implies suppression of the anti-inflammatory IL4/IL13 axis [32]. Janus kinase 1 (JAK1) and 5-lipoxygenase activating protein (FLAP) play pivotal roles in respectively, cytokine receptor signalling and biosynthesis of eicosanoids (lipid mediators of inflammation). Galectins (Fig. 5) are carbohydrate binding proteins involved in various immune processes [33]. Notably, expression of a panel of immune genes decreased by day 30.

**Figure 9:**
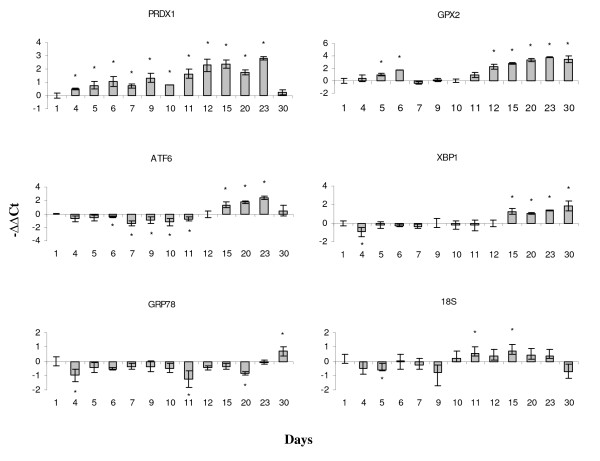
**Genes for antioxidants and proteins involved in unfolded protein responses analyzed with real-time qPCR**. PRDX1 - peroxiredoxin 1, GPx2 - glutathione peroxidase 2, ATF6 - activating transcription factor 6, XBP1 - x-box binding protein 1, GRP78 - glucose-regulated protein 78, 18S.

The most noticeable aspect of the last stage of adipogenesis was co-ordinated activation of genes involved in various stress responses. Several components of the activator protein complex 1 (AP1) (c-Fos, c-Jun, JunB1, JunB2, and JunD), which co-ordinates responses to pathogens and stressors, showed highest expression levels at day 30 (Fig. 5). The observed gene expression changes clearly revealed the endoplasmic reticulum (ER) stress in our culture at this stage. The up-regulated oxidant stress-activated serine/threonine kinase 25 (YSK1, Fig. 5) is a Golgi complex-associated regulator of transport of proteins and lipids to plasma membrane [34]. The A-kinase anchor protein 9 (AKAP9) is an essential mediator in lipolytic pathways and is necessary for the maintenance of Golgi structure through interactions with signalling proteins, including the protein kinase N (PKN1); both AKAP9 and PKN1 showed strong down-regulation at day 15 (Fig. 5). The ER stress response involves a set of mechanisms referred to as unfolded protein response (UPR) [35]. The classical UPR markers X-box-binding protein 1 (XBP1) and activating transcription factor 6 (ATF6) were up-regulated during the lipid-loading phase of adipogenesis (Fig. 9). Our findings point to the increased responses to oxidative stress with time. The glutathione peroxidases (GPXs) (Fig. 5 and 9), which are sensitive to lipid mediated peroxidation [36], were similarly induced and a suite of genes coding for proteins of glutathione metabolism also had high levels, at the time when cells engaged in increased LD formation and expansion. The thioredoxin (TXN) antioxidant system is also involved in the regulation of intracellular ROS [37]. The production of ROS is catalyzed with iron and hem, therefore, the up-regulation of ferritin (the intracellular iron storage protein), and two key enzymes in the metabolism of hem, aminolevulinate δ synthetase and heme oxygenase 2 (HMOX2) suggest the induction of mechanisms that protect against oxidative stress at day 30.

**Figure 10:**
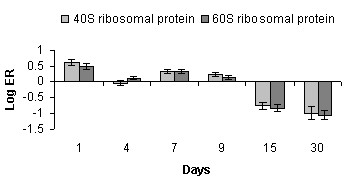
**Ribosomal proteins analyzed with microarrays**. Data are mean log-(ER) ± SEM of twenty and ten genes involved in 60S and 40S subunit assembly respectively.

The massive down-regulation of components of the translational machinery and translation initiation factors suggested reduction of ribosomal biogenesis and attenuation of protein translation while the highest expression level of 18S at day 15 suggested profound changes in the composition of ribosomes during terminal differentiation (Fig. 5, 9 and 10). Averaged expression profiles of thirty five highly co-expressed ribosomal proteins, components of the 40S and 60S ribosomal subunits, were shown in Fig. 10. Interestingly, eukaryotic initiation translation factor 5 (eIF5) involved in the joining of ribosomal subunits resulting in the formation of a functional 80S initiation complex was up-regulated at the last time point (Fig. 5).

The increase of several protein folding heat shock proteins and genes involved in protein degradation was evident during this period (Fig. 5 and 10). The components of the 26S proteasome, ubiquitin and several enzymes involved in the ubiquitin conjugation to target proteins were up-regulated at day 15 and even more markedly at the end of the study period. High levels of 26S proteasome non-ATPase regulatory subunit 14 (POH1), were observed at day 30 while up-regulation of lysosomal proteases (cathepsins) was seen from day 15 (Fig. 5).

## Discussion

Our transcriptomic analyses suggest, as expected, that early salmon aSVF culture contains a number of cell types, including vascular cells, macrophages and lymphocytes, in addition to preadipocytes. Similar cell composition of aSVF was reported in mammals [38]. The gene expression profiles at days 1 and 4 implied retention of multipotency of aSVF cells during the early stages of the culture. Decrease in gene expression of markers of osteo/chondrogenic, myogenic, immune and vasculature cell lineages (Fig. 5) and increase in adipogenic markers indicate that the preadipocyte precursors, probably due to their active proliferation, become the preponderant cell type at confluence.

Recent studies in mammals showed that tissue-resident MSCs originate from the smooth-muscle-like pericytes [39,40], which are laid over junctions of endothelial cells in blood vessel walls [41]. Once liberated from the endothelial cell, pericyte is activated and can be considered a stem cell [42]. Characterisics of the WAT population of stem cells and early events in the determination phase are much less studied than the terminal differentiation phase of adipogenesis. The breakthrough in the understanding of elusive origins of the adipogenic MSCs was made only recently in a study of Tang et al. [9], which demonstrated that pericytes surrounding blood vessel walls in WAT are precursor cells of preadipocytes. Gradual attenuation of the vasculature specific genes glomulin and LIVE1 and increase of the pericytic marker TAGLN at days 7 and 9 support perivascular origin of salmon preadipocytes as well. Early PPARγ expression could represent an additional evidence of the pericytic identity of adipocytes' precursors in our model since Tang et al. confirmed PPARγ as a specific marker of the perivascular fraction in subconfluent aSVF [9]. Indeed, our finding of high levels of PPARγ mRNA well before confluence contradicts the cell culture studies reviewed in [18] as the expression of C/EBPβ and C/EBPδ predate the expression of PPARγ and C/EBPα [17,18], which then activate adipocyte-specific genes during terminal differentiation. This illustrates the advantage of primary cell cultures, which offer possibilities to gain valuable insight into the early molecular events of adipogenic differentiation.

Commitment of multipotent MSCs to the adipogenic lineage involves simultaneous inhibition of other mesenchymal lineages. Starting from day 6 we observed consistently decreased expression of MyoD, an early marker of the myogenic lineage. MyoD regulates the cell cycle in terminally differentiated myocytes by inducing CDK inhibitor p21, which irreversibly arrests proliferation [43]. MyoD may play a similar role in mature adipocytes and in theory this could account for the increased expression at day 30. Activation of BMP4 from day 9 was an additional evidence for the inhibition of myogenesis. This growth factor equally promotes differentiation of the adipose, cartilage or bone lineages depending on the culture conditions [44,45]. Therefore, activation of BMP4 is necessary but not sufficient for the commitment of preadipocytes. PPARγ is a master adipogenic regulator, which simultaneously inhibits myogenesis, osteogenesis and chondrogenesis [44,46,47]. Thus, high expression of PPARγ observed long before confluence with the peak at day 6 probably posed a barrier to the induction of non-adipogenic mesenchymal cell lineages. Non-adipogenic developmental pathways are expected to be strongly suppressed by the addition of hormones. Yet, we did not find any noticeable changes in the markers of different cell types after day 9 and earliest increased level of the anti-osteogenic GAS6 was observed at day 15.

Our PCNA results suggested the highest proliferative activity during the first days of culture followed by a moderate reduction of mitotic activity at day 6 and much greater decrease by day 30. Overall, the expression patterns of genes involved in cell cycle were in concordance with these changes. Highest levels of the key negative cell cycle regulators (e.g. RBL1, p53-like, GADD45γ) were observed during days 15-30. An additional evidence for the anti-proliferative status was the up-regulation of several pro-apoptotic genes, including retinoic acid-regulated apoptosis-related protein 3 (APR3). APR3 arrests cell cycle by suppressing the activity of cyclin D1 [48], which was also induced at the latest stage. Differentiation of adipocytes in mammals is associated with a reduction in proliferation at confluence followed by subsequent activation or clonal expansion and eventually with cell cycle arrest at terminal differentiation [49]. Our results revealed the diversity of mechanisms potentially employed in the execution of the similar stage of events that occur during Atlantic salmon adipogenesis. Given that WAT is a highly specialized organ that functions to regulate metabolic homeostasis and energy balance, nutrient sensing mechanisms could be opted for a role in the control of proliferation. Growth cycle arrest of salmon adipocytes was in parallel with the up-regulation of the fatty acid binding protein 3 (FABP3) that participates in the uptake, intracellular metabolism and transport of long chain fatty acids. FABP3 seems like a good candidate that could be involved in this type of regulation since its growth arrest activity was previously demonstrated in mammalian epithelial cells [50]. Further, minimizing protein synthesis may also control proliferation as cell cycle arrest is a well established consequence of the general translational arrest, implied from day 15 onwards.

Microarray analyses suggested changes in nutrient metabolism in the late phase of adipogenesis (day 30). As a consequence of the up-regulation of pyruvate dehydrogenase kinase that inactivates pyruvate dehydrogenase, the Krebbs cycle most likely relied on the preferential use of acetyl-CoA from FA oxidation rather than pyruvate to cover the cellular energy requirements. Also, the expression of genes involved in FA β-oxidation, acyl-Coenzyme A dehydrogenase and acyl-Coenzyme A binding protein, steadily increased towards the end of adipogenesis. The up-regulation of G6PD and PGD pointed to the importance of the PPP, which generates NADPH required for accumulation of lipids. The PPP can be seen as an alternative to glycolysis, because in addition to generating NADPH and R5P, it can also provide cells with G3P, the phosphorylated glycerol backbone suitable for TAG synthesis. Induction of glycolytic PFK was also in line with the increased production of G3P and TAG biosynthesis. Biosynthesis of carbohydrates in carnivourous fish species could be important due to the very low carbohydrate dietary level in their natural diets [51]. Indeed, we have seen regulations in all major glucose metabolic pathways in our model (Fig. 8); however, differentiation of adipocytes did not involve induction of HK, one of the key enzymes of glucose metabolism that only showed slight up-regulation at day 30. This indirectly suggested that the transformation of gluconeogenic amino acids into glucose is important in adipocytes of Atlantic salmon, at least *in vitro*. In addition, late activity of two key glyceroneogenic genes, MDHC and PEPCKC implied that glyceroneogenesis, which can be fed with mitochondrial intermediates derived from lactate and gluconeogenic amino acids, in addition to pyruvate, was active in developing fish adipocytes. To conclude, these findings illuminate potentially novel aspects of nutrient metabolism in adipocytes, which might take advantage of the coupling of glycolysis, the PPP and glyceroneogenesis, in order to fulfil shifting demands for the three major products of these pathways: G3P, R5P and NADPH.

NADPH produced by PPP is also crucially important for the cellular antioxidant defence since it is required for the regeneration of oxidized glutathione by glutathione reductase (GSR). Up-regulation of GSR and a suite of genes involved in metabolism of glutathione, thioredoxin and iron was a prominent feature of late salmon adipocyte differentiation (days 15-30). Indeed, a recently introduced concept [52-54,4] clearly demonstrated ROS as anti-adipogenic molecules that inhibit preadipocyte proliferation and differentiation. A reduced redox state in WAT is now recognised as a distinct characteristic of visceral obesity in mammals, with overexpression of GPX peroxidases and high content of hydrophilic antioxidant gluthathione associated with pro-adipogenic processes. Induction of the number of GPXs, known to be sensitive to lipid peroxidation, implied ROS-provoked response in our culture and the need to maintain highly reduced state of the intracellular environment. However, contrary to what could be expected in situations of high ROS production, neither gene expression nor activity of SOD increased. In fact, the activity of this important ROS scavenger progressively declined during adipocyte differentiation. Thus, our data corroborated the indespensible role of glutathione-based antioxidant system in the maintenance of the reduced intracellular state in white adipocytes of fish.

Accumulation of lipids in adipocytes coincided with the activation of genes coding for secreted proteins, such as adipokines adipsin and visfatin. Together with the previous finding of leptin secretion by Atlantic salmon adipocytes [10], this supports the notion of WAT as an active endocrine organ in fish. Enhanced secretion may impose an additional load on the ER actively engaged in lipid droplet synthesis [55]. The ER compartment is suspected to exert a great deal of control over adipogenesis and association of obesity with ER-stress is firmly established [56]. Up-regulation of XBP1 and ATF6 at day 15 in salmon adipocytes implied activation of UPR in response to perturbations in the ER homeostasis [57]. Moreover, endoplasmic GRP78, which is located downstream from ATF6 in the UPR signalling cascade was down-regulated throughout the whole studied period but increased in expression at day 30. A number of changes that are typical of UPR [35] was a hallmark of the latest stages of adipogenesis in our model. UPR as a collection of pathways aimed at restoring ER function may serve different adaptive roles and is observed both under pathological and normal physiological situations [58]; for example, it is part of the developmental program in highly specialized secretory B and T cells [59] and ß-pancreatic cells [60]. The revealed UPR in developing fish adipocytes appears as a highly tailored homeostatic mechanism that is activated when increased number and size of LDs meat the limited ER capacity. Although ER is mainly considered to be a protein-folding factory, all proposed models of LD formation emphasize the engagement of the ER compartment in the process [61].

UPR utilises two broad strategies to relieve stress in the ER: increased clearance of misfolded proteins from the ER and reduction of new protein influx into the ER. We found ample evidence for the activation of the 26S proteasome and autophagy-lysosomal proteolytic pathways, which degrade misfolded proteins. Components of the membrane protein complex, derlin 1 and p97-interacting cofactor p47, which mediate transport from the ER lumen into the cytosol were induced at day 30, while other proteolytic pathways were up-regulated even earlier. UPR-induced proteolysis may be employed to initiate the breakdown of superfluous proteins and organelles that must be replaced by more highly specialised cellular components during the adipocyte development. Without a doubt, considerable remodelling of the cellular architecture occurs during terminal differentiation of Atlantic salmon adipocytes.

UPR also activates genes involved in protein folding. Hsp40s and other J-domain-containing proteins act as co-chaperones for Hsp70s helping to restore homeostasis in the ER and in addition may have pro-degradation roles. Down-regulation of translational machinery is commonly observed during UPR [35], and in our study, it involved a dramatic reduction in expression of ribosomal proteins.

Alteration of the ribosomal composition is most certainly involved in the multiple regulation of the ER-stress response. The curious up-regulation of the 40S subunit 18S rRNA through most of the terminal differentiation against the majority of down-regulated ribosomal proteins is bound to have important consequences given the high level of 18S constitutive expression. This could signify a switch to a less efficient protein translation as increase in 18S negatively affects the ribosomal subunit ratio that favours protein production [62]. On the other side, reduced expression of 18S at the very end of differentiation could improve the effectiveness of protein synthesis in adipocytes that acquire secretory phenotype. In conclusion, fully matured adipocytes seem to reprogram the pattern of gene expression to sustain a certain level of protein production, congruent with the now recognised endocrine functions of WAT.

Secreted adipsin and visfatin have multiple immune functions as most other adipokines [63]. Overall, expression changes of a large number of immune genes were characteristic of Atlantic salmon aSVF culture. Dissimilar temporary profiles suggest different regulatory and physiological roles of these genes. Additionaly, high initial levels and subsequent decrease of the chemokine receptors, TNFα and a panel of TNF-related genes could be explained by the changes in relative abundance of non-adipogenic cells that possess pronounced immune properties. In mammals, TNFα has high anti-adipogenic activity [64], which was recently shown to be conserved in fish [65]. Therefore, down-regulation of the TNF axis at days 7-9 was probably important for the onset of adipocyte differentiation. However, a number of immune genes showed either stable increase since day 4 (complement component C5) or biphasic regulation with the second activation after hormone induction (LECT2). Highest expression levels of many genes with diverse roles in different inflammatory pathways were observed during terminal adipocyte differentiation. Numerous independent studies have provided evidence that a set of inflammatory and stress-response genes is activated in obesity in mammals (reviewed in [66]). Some of these genes likely have important roles in the coordination of homeostasis in WAT, similar to the established roles of TNFα in modulating proliferative abilities in preadipocytes or metabolic activities in mature adipocytes.

An intriguing example is a group of virus responsive genes including TLR3, a receptor of double stranded RNA and a group of galectins, previously not reported to have roles in any aspect of adipocyte biology. The latter includes galectin 9, which showed highly specific responses to viruses in previous studies from our group [67,68]. However, a large portion of immune genes, including complement component C5, JAK1, the key actor of interferon signaling and FLAP, the regulator of eicosanoid metabolism, decreased expression by the time of AP1 establishment. Finally, our results do not permit for major conclusions as to whether immune genes are important for adipogenesis or change expression as a consequence of differentiation and/or stress responses. However, it is clear that Atlantic salmon WAT possesses high potential for immune activity.

It is tempting to explain the activation of immune genes in differentiating adipocytes as a side effect of adaptation to the oxidative and ER stress, since stress and immune responses share common regulatory pathways. Ozcan et al. [56] showed that ER-stress-induced JNK-AP1 axis is the central link between TAG overload in liver and diabetes. Most interestingly, this is in agreement with our study, which shows that the same players are activated in fish adipocytes in response to increased lipid deposition. Continuous administration of lipids led to the maximal observed TAG loading at the end of the studied period, when the collective and highest expression of the AP1 complex components, including all c-Jun and JunB members was also observed. This additionally coincided with the induction of POH1, a regulatory subunit of the 26S proteasome that has a specific de-ubiquitinase activity towards c-Jun leading to its accumulation and subsequent increase in AP1-mediated gene expression [66]. A suite of highly expressed immune genes at this time supported an inflammatory type reaction in adipocytes. Hence, an unrelieved ER-stress could likely be the major cause of chronic inflammatory responses in lipid overloaded white adipocytes in fish.

## Conclusions

Our study revealed concordance between the gene expression profiles and the key events during adipogenic development of the primary culture of aSVF cells, including fluctuations of proliferative activity, induction of adipogenic differentiation and suppression of other mesenchymal cell lineages and final tuning of metabolism towards production and accumulation of lipids. For the first time in a fish species we show that the establishment of mature adipocyte phenotype is associated with high activity of immune genes, activated UPR and responses to oxidative stress. These changes are likely to be part of the normal adipocyte development but may be accentuated when cells are overloaded with lipids. High expression of pro-inflammatory mediators imply that excessive growth of WAT in fish may cause disturbed endocrine function with possible negative effects on health, as seen in mammals.

## Methods

### Preadipocyte isolation and culture conditions

Atlantic salmon was reared at Nofima Marin station at Averøy, Norway on a commercial diet to weight of 2-3 kg. Fish were anaesthetized with metacain (MS-222), bled by cut of arch bows of the gills and killed by a blow to the head. The experiment was conducted according to the National Guidelines for Animal Care and Welfare of the Norwegian Ministry of Research. Visceral adipose tissue was excised and salmon preadipocytes were isolated as described in Vegusdal et al. [10]. Briefly, the dissected fat tissue was washed with phosphate buffered saline (PBS, pH 7.4) (unless otherwise stated, all chemicals were obtained from Sigma-Aldrich Chemical Co., St. Louis, MO, USA), minced and digested in 0.1% collagenase (type I) in HBSS (1 g tissue/5 mL HBSS) at 13°C for 1 h under shaking and filtered through 250 and 100 μm nylon. The resulting cell suspension was then centrifuged at 700 × *g *for 10 min at 10°C. The buoyant fat layer with mature adipocytes was removed by aspiration, while the preadipocytes were pelleted on the bottom. After washing twice, the cells were resuspended in growth medium containing L-15, 10% fetal bovine serum (FBS), 2 mM L glutamine, 10 mM HEPES, and antibiotics (mixture of penicillin, streptomycin and amphotericin B) and seeded on laminin coated cell-culture flasks at a density of approximately 10 g tissue/25 cm^2^. The cells were kept at 13°C and media were changed every 3 days. Cells reached confluence after approximately 1 week. Confluent cells were cultivated for 48 h in an differentiation inducing medium, i.e. growth medium supplemented with 1 μM dexamethasone, 33 μM biotin, 10 nM triiodothyronine, 17 μM panthothenate and 25 μM isobutylmethylxanthine, 20 μg/ml insulin and a lipid mixture (1 μl/ml; corresponding to 45 mg/ml cholesterol, 100 mg/ml cod liver oil FA (methyl esters). After that cells were transferred to a maintenance differentiation media containing growth media only supplemented with 2 μl/ml of lipid mixture. Media was changed every 3 days until the cells reached the final differentiation stage with morphology of mature adipocytes (day 30).

### Electron microscopy

Cells for electron microscopy studies were taken from cultures at days 15 and 30. Cells were washed in 0.1 M PBS (pH 7.4), then fixed in 2% glutar aldehyde in 0.1 M cacodylate buffer (pH 7.4) at 4°C for 24 h. The cells were the harvested, rinsed in 0.1 M cacodylate buffer and post-fixed for 60 min in 2% OsO_4 _containing 1.5% potassium ferrocyanide, followed by en bloc staining with 1.5% uranyl acetate. Cells were dehydrated in a series of ethanol solutions (70%, 90%, 96%, and 100%) and propylene oxide, and then embedded in epon resin, which was polymerized at 60°C for 24 h. Ultrathin sections (approximately 50 nm) were cut on a Reichert Ultracut E ultramicrotome using a diamond knife. The sections were placed onto formvar/carbon-coated 75-mesh copper grids, post-stained for 2 min with 0.2% lead citrate solution in 0.1 M NaOH, and examined in a Philips CM 100 transmission electron microscope at an accelerating voltage of 80 kV.

### Microarray analyses

The samples for microarrays analyses were taken at days 1, 4, 7, 9, 12 and 30. The salmonid fish microarray (SFA2, immunochip) includes 1800 unique clones printed each in six spot replicates. Total RNA was extracted by using RNeasy^® ^Mini Kit (Qiagen, Valencia, CA, USA), according to the manufacturer's instruction. RNA was treated with RNase-free DNase I (Qiagen, Valencia, CA, USA), to remove any contaminating DNA. All RNA samples used in our experiments had A260/280 ratios between 1.80 and 2.30. The total RNA concentration was determined at 260 nm using spectrophotometry. Equal inputs from 5 cell flask replicates were pooled for each time point. Equalised control was made by mixing RNA from each time point. The test and control samples (15 μg RNA in each) were labelled with respectively Cy5-dUTP and Cy3-dUTP (Amersham Pharmacia, Little Chalfont, UK). The fluorescent dyes were incorporated in cDNA using the SuperScript™ Indirect cDNA Labelling System (Invitrogen, Carlsbad, CA, USA). The cDNA synthesis was performed at 46°C for 3 h in a 20 μl reaction volume, following RNA degradation with 0.2 M NaOH at 37°C for 15 min and alkaline neutralization with 0.6 M Hepes. Labelled cDNA was purified with Microcon YM30 (Millipore, Bedford, MA, USA). The slides were pretreated with 1% BSA fraction V, 5 × SSC, 0.1% SDS (30 min at 50°C) and washed with 2 × SSC (3 min) and 0.2 × SSC (3 min) and hybridized overnight at 60°C in a cocktail containing 1.3 × Denhardt's, 3 × SSC 0.3% SDS, 0.67 μg/μl polyadenylate and 1.4 μg/μl yeast tRNA. After hybridization slides were washed at room temperature in 0.5 × SSC and 0.1% SDS (15 min), 0.5 × SSC and 0.01% SDS (15 min), and twice in 0.06 × SSC (2 and 1 min, respectively). Scanning was performed with Axon GenePix 4100A and images were processed with GenePix 6.0 (Molecular Devices, Sunnyvale, CA, USA). The spots were filtered by criterion (I-B)/(SI+SB) ≥ 0.6, where I and B are the mean signal and background intensities and SI, SB are the standard deviations. Low quality spots were excluded from analysis and genes presented with less than three high quality spots on a slide were discarded. After subtraction of median background from median signal intensities, the expression ratios were calculated. Lowess normalization was performed first for the whole slide and next for twelve rows and four columns per slide. The differential expression was assessed by difference of the mean log-expression ratios between the slides with reverse labelling (6 spot replicates per gene on each slide, Student's t-test, p < 0.01). Complete microarray results are submitted to NCBI GEO Omnibus (GSE18389).

### Quantitative real-time RT-PCR

RNA isolated at days 1, 4, 5, 6, 7, 9, 10, 11, 12, 15, 20, 23 and 30 was used for qPCR. Approximately 200 ng of total RNA was reverse-transcribed into cDNA using TaqMan^® ^Gold RT-PCR Kit (Applied Biosystems, Foster City, CA, USA), a 25 μl reaction system according to the manufacturer's protocol. The PCR primers (Table 1) were designed using the Vector NTI (Invitrogen, Carlsbad, CA, USA) and synthesized by Invitrogen. Efficiency was checked from tenfold serial dilutions of cDNA for each primer pair. A 2 × SYBR^® ^Green PCR Mastermix (Roche Diagnostics, Mannheim, Germany), 0.4 μM of each primer, and the cDNA template were mixed in 12 μl volumes. PCR was performed in duplicates in 96-well optical plates on Light Cycler 480 (Roche Diagnostics, Mannheim, Germany). Different controls were used for microarray and qPCR. All time points were compared relative to day 1 in qPCR analyses. Relative expression of mRNA was calculated using the ΔΔCt method. Three commonly used genes (18S, elongation factor 1A and eukaryotic translation initiation factor 3, subunit 6) were tested for stability using the GeNorm and NormFinder. Finally, elongation factor 1A (EF1A) met criteria of stability in the analyzed material. Differences between control and cells at different developmental stages were assessed with Student's t-test (p < 0.05).

**Table 1:** Primers used for real-time qPCR analyses

Gene	Forward primer (5'-3')	Reverse primer (5'-3')	Accession number
Tumor necrosis factor alpha (TNFα)	AGGTTGGCTATGGAGGCTGT	TCTGCTTCAATGTATGGTGGG	AF321836
Tumor necrosis factor (TNF) decoy	TCTCCTGGTATTTGCGCTCTGTGGT	TATAAGTCGGTGTGTGAGCGCCTGA	CA351440
Arachidonate 5-lipoxygenase-activating protein (FLAP)	TCTGAGTCATGCTGTCCGTAGTGGT	CCTCCCTCTCTACCTTCGTTGCAAA	CA369467
Tyrosine-protein kinase 1 (Jak1)	GAGGAGTTTGTCCAGTTCGGTCCGT	CATGCACCAGCTTCTTATCCTCCAG	CA368994
Programmed death ligand 1 (PD-L1)	TCAACGACTCTGGGGTGTACCGATG	TCCACCTCATCTCCACCACGTCTC	CA366631
Interleukin 13 receptor alpha 2 (IL13Rα2)	TCTCTGAGCCGCTCAACCTGTCAT	CGTTCCACGACAGCTTTATACGGA	CA348044
Leukocyte cell-derived chemotaxin 2 (LECT2)	CTGTGTTGTCAGAGTGCGAGATGGT	TACACACAATGTCCAGGCCCTGA	EXOB2
Complement component 5 (C5)	AGAACTCTTCCGAGTTGGCATGGT	AGTGATGCTGGGATCCATCTCTGA	CA364804
Proliferator-activated receptor gamma (PPARγ)	CGTGTATCAAGACGCCAGCT	TTGCAGCCCTCACAGACATG	EU655708
Bone morphogenic protein 4 (BMP4)	TCAAGTTGCCCATAGTCAGT	CACCTGAACTCTACCAACCA	FJ195610
Myogenic factor D (MyoD)	CCGCAACACGAAGCAACTATTACAGC	GGAACCCTCCTGGCCTGATAACAC	AJ557150
CCAAT-enhancer-binding protein beta (C/EBPβ)	CAAACTACATTACCAGGC	GTTATGTGTTGCCAGTTG	EU668996
CCAAT-enhancer-binding protein delta (C/EBPδ)	TTGGGCGGTGGAGCCTAT	TTTCCTCGCCCGTGTCAT	EU668997
CCAAT-enhancer-binding protein alpha (C/EBPα)	AGACCTCGGCGAGATTTGT	TGTGGAATAGATCAGCCAGGAA	EU668995
Hexokinase (HK)	GCTGAAGACCAGAGGCATCTTTGA	GCTGCATACCTCCTTGACGATGAT	AY864082
Glucose-6-phosphate dehydrogenase (G6PD)	TGGTGCAGAACCTCATGGTCCTCA	ATCCCGGATGATTCCAAAGTCGTC	BT044902
Phosphofructokinase (PFK)	AATCCATCGGCGTTCTGACAAGC	GCCCGTACAGCAGCATTCATACCTT	BT059256
Pyruvate kinase (PK)	TGCCTTCATTCAGACGCAGCA	CAGATGATTCCGGTGTTGCGA	BT043851
Malate dehydrogenase cytoplasmic (MDHC)	AGACGTCCACCACTGCAAGGTCAA	TTAACAGCGTCGAAGCAGGCCA	BT043497
Cytosolic phosphoenolpyruvate carboxykinase (PEPCKC)	AGGGCATGGACCAGGAACTCC	GGGCTCTCCATCCTGGGATGT	BT072418
6-phosphogluconate dehydrogenase (PGD)	CCAATGAGGCTAAAGGCACCAAGA	CCAGCTTGTCGATGAAGTCATCCA	BT050391
Transketolase (TKT)	TGCCATCTCCGAGAGCAACATC	CCGTGGGAATGGCTCTGAACAT	BT059642
Peroxiredoxin 1 (PRDX1)	CACTGCTGTGGTGGATGGACAGTT	CCAGCGGGTAGAAGAAGAACACCA	est02b08
Glutathione peroxidase 2 (GPX2)	TGTACCTCAAGGAGAAGCTGCCGT	ATTAAGGCCATGGGATCGTCGC	est04e05
Activating transcription factor 6 (ATF6)	CTCACACCATCAAAGCTACAGCGA	GTGTCGCCTCGTCGATTTAACTCA	CA367172
X-box binding protein 1 (XBP1)	CGACTCAAATTCGACAACTGGGC	TTTCTGTCTCTGGCTGTCTGGGCT	CA385697
Microsomal triglyceride transfer protein (MTP)	CAAAGACCAGCGTCAACAACAA	CGCCTCTGTCTCAAAGCTCACT	CA042356
78 kDa glucose-regulated protein (GRP78)	GTGCAGCATGACATCAAGTA	CTCTTCTTCTCGATAACCTT	CA368961
Cluster of differentiation 36 (CD36)	GGATGAACTCCCTGCATGTGA	TGAGGCCAAAGTACTCGTCGA	AY606034
ATP-binding cassette transporter 2	AGCGGGGGAAACAGTAGCAGGA	GCCTGGTCTTGAGATTGTGGGTGT	CA343913
Lipoprotein lipase (LPL)	TGCTGGTAGCGGAGAAAGACAT	CTGACCACCAGGAAGACACCAT	BI468076
Fatty acid(FAS)	TGCCTCAGCACCCTACTCTG	GCTTTACAACCTCAGGATTGGC	BT048827
18S	TGTGCCGCTAGAGGTGAAATT	GCAAATGCTTTCGCTTTCG	AJ427629
Elongation factor 1A (EF1A)	TGCCCCTCCAGGATGTCTAC	CACGGCCCACAGGTACTG	BG933853
Eukaryotic translation initiation factor 3 subunit 6	GTCGCCGTACCAGCAGGTGATT	CGTGGGCCATCTTCTTCTCGA	CX040383

### Cell proliferation

Cell proliferation was assessed by the immunocytochemical detection of PCNA)(Zymed Laboratories, South San Francisco, CA, USA). Cells were washed in PBS and fixed in 70% ethanol for 30 minutes at 4°C. Endogenous peroxidise activity was blocked with with 3% hydrogen peroxide in methanol for 10 min. The cells were washed three times with in PBS, then incubated with a mouse anti PCNA monoclonal antibody (clone PC10) using a PCNA immuno detection kit, following the manufacturer's instructions. The cells were counterstained with Mayer's haematoxylin for 2 min, washed in water, dehydrated in a graded series of alcohol solutions, cleared with xylene, and mounted with Histomount. PCNA-containing nuclei were stained dark brown. Two hundred cells were observed and the percentage of proliferating cell was calculated.

### Lipid accumulation in adipocytes

The quantity of lipids in cytoplasm were estimated by oil-red O staining according to Ramirez-Zacarias et al. [70] on days 1, 4, 7, 9, 15 and 30. Briefly, cells were washed twice with PBS, fixed with 10% cold formalin for 30 min, rinsed in water and stained for 2 h with filtered oil red O in isopropanol at room temperature. For relative quantitative measurements of TAGs accumulation, bottles were washed with PBS to remove excess of stain solution, dried, the colour was dissolved in 100% isopropanol and the absorbance was measured spectrophotometrically in a Victor 3 microplate reader PerkinElmer (Wellesley, MA, USA) at 500 nm.

### Superoxide dismutase assay

SOD activity was assayed with a kit (Cayman Chemicals, Ann Arbor, MI, USA), which utilizes a tetrazolium salt to detect superoxide radicals generated by xanthine oxidase and hypoxanthine. Colour was measured at 405 nm in a Titertek Multiskan PLUS MKII (Labsystems, Helsinki, Finland) plate reader. One unit of SOD was defined as the amount of enzyme needed to achieve 50% dismutation of the superoxide radical.

## Authors' contributions

MT and BR contributed to the overall experimental design. SS and AK carried out microarray analyses. MT carried out cell culture work, and assay analyses. MT and SS carried out qPCR analyses and produced the first manuscript draft. All authors read, contributed to, and approved the final manuscript.
